# Analysis of an Association between Preterm Birth and Parental Educational Level in Japan Using National Data

**DOI:** 10.3390/children10020342

**Published:** 2023-02-09

**Authors:** Tasuku Okui

**Affiliations:** Medical Information Center, Kyushu University Hospital, Fukuoka City 812-8582, Japan; okui.tasuku.509@m.kyushu-u.ac.jp; Tel.: +81-092-642-5881

**Keywords:** Japan, preterm birth, educational attainment, vital statistics

## Abstract

Preterm birth rate depending on parental educational level in recent years has not been surveyed in Japan. In this study, we showed the trend in preterm birth rate depending on parental educational level from 2000 to 2020 by linking data from the Census regarding individuals’ educational level and parents in birth data of the vital statistics. Four types of parental educational level, namely junior high school, high school, technical school or junior college, and university or graduate school, were compared. Slope and relative indexes of inequality for preterm birth by educational level were computed by binomial models. Data on 3,148,711 births and 381,129,294 people were used in the analysis, and data on 777,086 singleton births were used after data linkage. The preterm birth rate (%) for junior high school graduate mothers and fathers was 5.07 and 5.21 in 2020, respectively. Contrarily, the preterm birth rate (%) for parents who graduated from a university or graduate school was 4.24 for mothers and 4.39 for fathers, and the rate tended to increase as educational level decreased, irrespective of parental gender. Results of inequality indexes showed that a statistically significant inequality by parental educational level persisted from 2000 to 2020.

## 1. Introduction

Preterm birth rate is one of the representative adverse birth outcomes, and it can affect early neonatal mortality or infant mortality [1,2]. Preterm birth rates are known to greatly differ between nations in the world [3], and Japan is recognized to have a comparatively low rate compared to other nations. Although preterm birth rate in Japan has increased since the late 20th century [4], it has remained constant in recent years [5].

Preterm birth rate is known to vary depending on sociodemographic factors, such as parental educational level, race, and income [6,7,8]. In Japan, some studies investigated socioeconomic factors related to preterm birth. Preterm birth rates are especially high in households without jobs, according to an analysis of national statistics [5]. An epidemiological study of a region in Japan using data from 2002 to 2013 indicated that the relative risk of preterm birth was significantly higher for infants whose father’s highest level of education was 13–15 years compared with 10–12 years [9], and an association was not demonstrated for maternal educational level. Another epidemiological study using data from 2008 to 2010 revealed that a lower educational level was associated with preterm birth [10]. Furthermore, a study employing national data of Japan demonstrated that lower parental educational attainment was associated with higher preterm birth rates [11]. In contrast, the study was carried out using data from 2001, and an association between preterm birth rate and parental educational attainment in more recent years has not been investigated yet in Japan using national data. It has been shown in Britain, Canada, the Netherlands, and the United States [12,13,14,15] that a discrepancy in poor birth outcomes based on socioeconomic characteristics fluctuated with time; therefore, it is crucial to determine whether or not a disparity was maintained in Japan. Furthermore, the proportion of university graduates is rising, and the younger generation in Japan is achieving higher levels of education [16]. Contrarily, the preterm birth rate has been rather steady in recent years [5], making it important to look into how the rate varies over time depending on educational attainment. 

The aim of this study is to show a trend in preterm birth rate by parental educational levels in recent decades in Japan and evaluate whether a disparity by parental educational levels persisted or not. 

## 2. Materials and Methods

### 2.1. Data Used in This Study

Individual-level birth data from vital statistics and the Census were employed for the analysis. The Ministry of Health, Labour, and Welfare of Japan as well as the Ministry of Internal Affairs and Communications provided the data. The data in 2000, 2010, and 2020 were used because the Census surveyed educational levels in those periods. In 2000, 2010, and 2020, there were 126,925,843, 128,057,352, and 126,146,099 people, respectively. 

The Census contains information on a person’s prefecture, municipality, household, gender, marital status, birth year, birth month, and degree of education. Educational level is classified as “currently studying at school” and graduates, and graduates are divided into junior high school, high school, “technical school or junior college”, and “university or graduate school”. The four types of graduates were compared in the analysis. 

The birth data for each infant comprises the infant’s birth year, prefecture, municipality, gender, parents’ birth year and month, parity, multiple births, mother’s age, household occupation, and gestational age. Preterm birth was defined as a birth whose gestational age was less than 37 weeks. Maternal age groups were categorized into <20 years, 20–24 years, 25–29 years, 30–34 years, 35–39 years, and ≥40 years. Parity was categorized into two types: primiparous and multiparous. Household occupation means the main occupation in the household, and it is categorized as self-employed, farmer, full-time worker 1, full-time worker 2, other occupations, or unemployed. Full-time worker 1 describes workers working in a company that has less than 100 employees, and full-time worker 2 describes other workers working in a company or as public servants. 

### 2.2. Data Linkage

Data linkage was performed to link individuals’ educational level data in the Census and parents’ birth data. Common ID does not exist in Japan, and deterministic data linkage was carried out using common information in both datasets, that is, birth year, birth month, gender, prefecture, and municipality. However, in this case, too many candidate men or women existed for parents of one birth. Therefore, we added two restrictions in the linkage. First, candidate men or women in the Census were restricted to married men and women to avoid the possibility that non-married men or women are matched with birth data of legitimate children. Additionally, parents of a single birth were matched only with men and women who reside in the same household because it is considered that the majority of parents giving birth do so. Only one-to-one matching pairs between parents of birth data and couples in the Census were used in the following analyses. As performed in earlier research [4,5], only singleton births were included in the analysis. Additionally, birth records involving parents who were studying at the time were not used.

**Figure 1:**
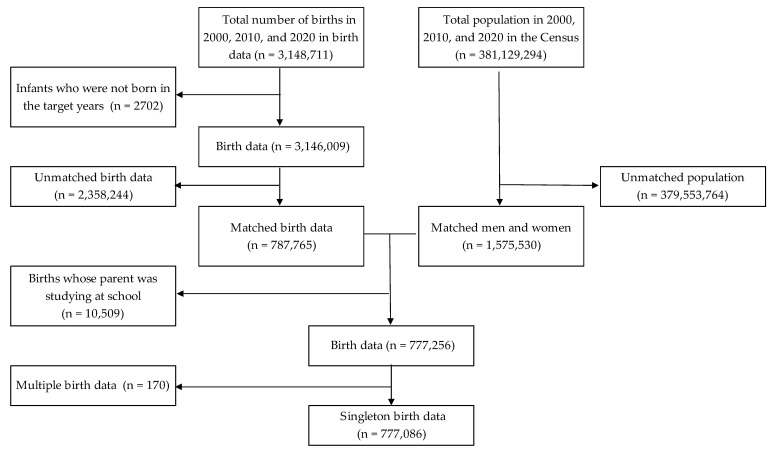
The flowchart of the data selection.

Figure 1 shows the flowchart of the data selection. In total, 3,148,711 babies were born in the years 2000, 2010, and 2020. Finally, data on 777,086 births were used.

### 2.3. Statistical Analysis

#### 2.3.1. Descriptive Analysis

The number of births for each characteristic was calculated for each year, and the preterm birth rate was calculated by year and parental educational levels. A trend test was performed to verify whether a rise in parental education levels would result in a drop in the preterm birth rate. 

#### 2.3.2. Inequality Indexes

Furthermore, the slope index of inequality and relative index of inequality was calculated to evaluate a disparity in preterm birth depending on parental educational level. The relative index of inequality is the rate ratio between the highest and lowest educational levels, whereas the slope index of inequality is the absolute difference (%) in preterm birth between the two educational levels [17]. A quantitative variable for educational levels ranging from zero to one was prepared for paternal and maternal educational levels. Specifically, the cumulative proportion of educational level was computed in hierarchical order, and the midpoint of the cumulative proportion for each educational level was used as the paternal and maternal educational level [18]. These variables for the educational attainment of fathers and mothers were incorporated into a regression analysis, and gender, parity, household occupation, and maternal age group were also adjusted. We made adjustments to these factors since they are linked to the preterm birth rate and their distribution possibly varied according to the educational attainment of parents. Specifically, a binomial regression model with an identity link was used to calculate the slope index of inequality with preterm birth as the outcome, and a log-binomial regression model was used to calculate the relative index of inequality [18,19,20,21]. We chose to employ a binomial model with an identity link because it allows us to calculate the absolute difference between the highest and lowest educational levels’ preterm birth rates. Furthermore, the log-binomial model was used because it calculates the ratio of preterm birth rates between people with the highest and lowest educational levels.

Although it was possible to use educational level as a categorical variable in a regression analysis, it is difficult to determine whether or not a disparity by educational level changed over time from the analysis. By using the disparity indexes, it becomes easier to compare degree of disparity over the years. Additionally, as it was revealed that the crude preterm birth rate tended to decrease with an increase in educational level linearly, we employed a method assuming a linear association between preterm birth rate and educational level. Furthermore, we included both the maternal and paternal educational levels in the regression model because one of them may be a confounding factor for the other. It is important to take into account of the influence of maternal educational level when evaluating the effects of paternal educational level.

#### 2.3.3. Other Points

As for the primary analysis, a complete case analysis was performed. Additionally, sensitivity analysis for missing data was performed, and hot-deck imputation was used as the imputation method [22]. All statistical analyses were performed using R, ver. 4.1.3 (https://www.R-project.org/ (accessed on 01 February 2023)). The authors’ analysis of national data yielded the statistical results presented in this paper, which differ from statistical data published by the Ministries.

## 3. Results

Table 1 depicts the number of births for each characteristic calculated for each year. The number of births in the analysis decreased over the years from 308,994 in 2000 to 216,637 in 2020 because of the decrease in births in Japan. The number of births from high school graduates was the largest in both fathers and mothers among educational levels, but the difference between high school graduates and the other educational levels decreased over the years.

Table 2 illustrates preterm birth rate by year and parental educational levels. Preterm birth increased from 2000 to 2010 and decreased from 2010 to 2020, regardless of educational level and gender. The preterm birth rate tended to increase as education levels declined, regardless of gender or year. In 2020, the percentage of junior high school graduates who had preterm births was 5.07 for mothers and 5.21 for fathers. Conversely, in 2020, the preterm birth rate (%) for parents who graduated from a university or graduate program was 4.24 for mothers and 4.39 for fathers. Trend test analysis revealed that the preterm birth rate statistically significantly increased with a decrease in educational levels (*p*-values < 0.05), regardless of the gender of the parents or the year.

In 2000, 2010, and 2020, the rank correlation coefficient between the quantitative variables for paternal and maternal educational level was 0.434, 0.436, and 0.422, respectively.

Table 3 shows the results of the slope index of inequality and relative index of inequality for preterm birth rate depending on educational level. The slope index of inequality was statistically significantly lower than zero both for paternal and maternal educational levels, regardless of year, and it indicates that preterm birth rate decreased as educational level increased, even when taking into account other risk factors (gender, parity, household occupation, and maternal age group). For instance, the slope index of inequality for paternal educational level in 2000 is −0.609, which shows a difference in the preterm birth rate between the educational levels with the highest and lowest levels. The relative index of inequality was also statistically significantly below one, and a similar result was obtained as the slope index of inequality. Moreover, the relative index of inequality for paternal educational level is 0.854, which indicates a ratio of preterm birth rates between educational levels with the highest and lowest levels. As a result, it was shown that inequality in preterm birth rate by parental educational level persisted over the years, and lower educational level was positively associated with higher preterm birth rate.

Results of the slope index of inequality and relative index of inequality for preterm birth rate depending on parental educational levels when applying an imputation approach are shown in Table S1 in the supporting information. Preterm birth disparity by parental educational attainment is shown as with the main analysis.

## 4. Discussion

In this study, we linked data from the Census and vital statistics and evaluated a disparity in preterm birth depending on parental educational levels. It was revealed that preterm birth rate tended to increase with a decrease in parental educational levels from 2000 to 2020. In addition, results of inequality indexes by regression models showed that an inequality in preterm birth depending on parental educational levels persisted from 2000 to 2020. A similar association between preterm birth and low parental educational levels has been shown in European countries and the United States in these periods [23,24,25]. In Japan, although results of an association between parental educational levels and preterm birth differed depending on studies in different periods [9,10], it was shown that a disparity persisted even in recent years using nationwide data. We discuss possible reasons for the result.

Regarding a possible link between lower educational level and preterm birth, smoking is one possible factor. An association between smoking and preterm birth has been shown in previous studies [26,27,28]. In Japan, the smoking rate is higher in persons with lower educational levels among both men and women [29]. Furthermore, women’s lower educational level was positively associated with a higher risk of gestational hypertension or diabetes [30,31,32], while the association with gestational diabetes is not conclusive [33]. These diseases are risk factors for preterm birth [34,35], and a higher rate of pregnancy complications in women with lower educational levels might be another reason for the association between preterm birth and lower educational level. Another possible factor for the association is the utilization of prenatal care. In other nations, prenatal care has been found to reduce the risk of preterm delivery [36,37,38], and antenatal care visits have been linked to educational level [39]. It is known that the proportion of insufficient utilization of prenatal care is high in areas where the high school enrollment rate is low, including in Japan [40]. 

Additionally, it was suggested that maternal socioeconomic position influences preterm birth directly, without the use of mediators [41]. Moreover, it is considered that fathers’ educational levels as well as mothers’ levels affects preterm birth [25]. It is known that income varies depending on the educational level in Japan [42], and it is considered that family income varies mainly by the father’s educational level because wages are higher for men compared with those of women. In the Philippines, the Netherlands, and the United States, it has been demonstrated that low income is linked to poor birth outcomes [43,44,45]. Furthermore, neighborhood deprivation levels have been linked to preterm birth [46,47], and factors such as healthcare accessibility, local crime rates, social cohesiveness, air pollution, greenness, and walkability have been suggested as potential mediators [46]. The discrepancy in preterm birth rates in Japan may be caused by a relationship between these socioeconomic characteristics and parental educational attainment.

The study results implied that more support and guidance for these high-risk people may be required during a pregnancy period. This has meaningful implications for the mechanism and reasons for the association between educational level and preterm birth in Japan. In addition, seeking a method to aid pregnant women with lower educational levels who do not fully utilize prenatal care is also needed. Increasing opportunities for education about pregnancy and prenatal care in schools or providing financial aid are possible methods. Furthermore, not only support from medical facilities but also workplaces and communities are important because the health behaviors of pregnant women are affected by the neighborhood environment [48].

We employed nationwide vital statistics data for the analysis, which is a strength of this study. In contrast, there are some limitations in this study. As a limitation of this study, the result is based on data linkage. Therefore, some errors, such as mismatches, might exist in the process of data linkage. For example, marital status may change in one year, and it is considered that some of the couples were not matched with birth data. In addition, many birth data could not be used in the analysis because they did not match with the Census data. Second, some essential maternal characteristics, such as body mass index, prenatal care use, and smoking habits, are not available within vital statistics data in Japan. Furthermore, there was a lack of information regarding medical comorbidities, antepartum infections, and neonatal variables such as congenital defects. To scrutinize the mechanism of the disparity, an epidemiological study surveying these factors is required. Third, this study is based on data on singleton births, and multiple births were not used.

## 5. Conclusions

In this study, we showed the trend in preterm birth rate depending on parental educational level from 2000 to 2020 using national data. As a result, it was shown that the preterm birth rate increased as educational level decreased, irrespective of parental gender and year. In addition, slope and relative indexes of inequality for preterm birth showed that a statistically significant inequality by parental educational level persisted from 2000 to 2020.

## Figures and Tables

**Table 1:** Number of births for each attribute by year.

	Year		
	2000	2010	2020
Total	308,994 (100.0)	251,455 (100.0)	216,637 (100.0)
Maternal age group			
19 years or less	9607 (3.1)	5076 (2.0)	2013 (0.9)
20–24 years	72,551 (23.5)	50,407 (20.0)	31,218 (14.4)
25–29 years	112,295 (36.3)	82,313 (32.7)	65,429 (30.2)
30–34 years	81,107 (26.2)	69,971 (27.8)	66,501 (30.7)
35–39 years	29,172 (9.4)	36,087 (14.4)	40,761 (18.8)
40 years or more	4262 (1.4)	7601 (3.0)	10,715 (4.9)
Gender			
Female	149,954 (48.5)	122,360 (48.7)	105,734 (48.8)
Male	159,040 (51.5)	129,095 (51.3)	110,903 (51.2)
Parity			
Primiparous	156,453 (50.6)	125,412 (49.9)	104,657 (48.3)
Multiparous	152,541 (49.4)	126,043 (50.1)	111,980 (51.7)
Household occupation			
Farmer	20,371 (6.6)	8193 (3.3)	4175 (1.9)
Self-employed	30,261 (9.8)	21,016 (8.4)	17,089 (7.9)
Full-time worker 1	116,984 (37.9)	96,872 (38.5)	75,969 (35.1)
Full-time worker 2	100,111 (32.4)	89,426 (35.6)	92,264 (42.6)
Other occupations	34,218 (11.1)	25,703 (10.2)	21,046 (9.7)
Unemployed	3624 (1.2)	3910 (1.6)	1721 (0.8)
Missing	3425 (1.1)	6335 (2.5)	4373 (2.0)
Paternal educational level			
Junior high school	36,536 (11.8)	21,616 (8.6)	13,555 (6.3)
High school	167,938 (54.3)	109,471 (43.5)	75,470 (34.8)
Technical school or junior college	34,399 (11.1)	34,600 (13.8)	27,607 (12.7)
University or graduate school	66,594 (21.6)	66,058 (26.3)	72,419 (33.4)
Missing	3527 (1.1)	19,710 (7.8)	27,586 (12.7)
Maternal educational level			
Junior high school	25,841 (8.4)	16,964 (6.7)	9896 (4.6)
High school	173,690 (56.2)	106,675 (42.4)	71,571 (33.0)
Technical school or junior college	83,233 (26.9)	72,275 (28.7)	54,595 (25.2)
University or graduate school	22,671 (7.3)	36,647 (14.6)	53,626 (24.8)
Missing	3559 (1.2)	18,894 (7.5)	26,949 (12.4)
Gestational age			
Term birth	294,936 (95.5)	239,867 (95.4)	206,784 (95.5)
Preterm birth	13,969 (4.5)	11,548 (4.6)	9821 (4.5)
Missing	89 (0.0)	40 (0.0)	32 (0.0)
Birthweight			
>= 2, 500 g	285,929 (92.5)	230,548 (91.7)	199,587 (92.1)
< 2500 g	23,042 (7.5)	20,876 (8.3)	17,023 (7.9)
Missing	23 (0.0)	31 (0.0)	27 (0.0)

**Table 2:** Preterm birth rate (%) by year and parental educational level.

	Year		
	2000	2010	2020
Total	13,597 (4.51)	10,246 (4.56)	8357 (4.52)
Paternal educational level			
Junior high school	1892 (5.27)	1045 (5.04)	686 (5.21)
High school	7446 (4.50)	4959 (4.68)	3366 (4.57)
Technical school or junior college	1439 (4.24)	1456 (4.33)	1187 (4.39)
University or graduate school	2820 (4.28)	2786 (4.32)	3118 (4.39)
Maternal educational level			
Junior high school	1397 (5.52)	854 (5.28)	488 (5.07)
High school	7834 (4.58)	4845 (4.72)	3248 (4.70)
Technical school or junior college	3438 (4.18)	3055 (4.35)	2388 (4.45)
University or graduate school	928 (4.13)	1492 (4.16)	2233 (4.24)

**Table 3:** Results of the slope index of inequality and relative index of inequality for the preterm birth rate depending on parental educational level.

	2000	2010	2020
	Estimates (95%CI)	Estimates (95%CI)	Estimates (95%CI)
Slope index of inequality			
Paternal educational level	−0.609 (−0.924, −0.293)	−0.620 (−0.976, −0.264)	−0.489 (−0.876, −0.103)
Maternal educational level	−1.024 (−1.344, −0.705)	−1.061 (−1.422, −0.700)	−0.967 (−1.353, −0.580)
Relative index of inequality			
Paternal educational level	0.854 (0.795, 0.918)	0.867 (0.800, 0.939)	0.886 (0.812, 0.967)
Maternal educational level	0.779 (0.723, 0.838)	0.773 (0.713, 0.839)	0.784 (0.719, 0.856)
CI, confidence intervals			
1. Gender, parity, household occupation, and maternal age group were adjusted in the analysis.			
2. Estimates for the slope index of inequality, which was calculated using a binomial model with an identity link function, can be interpreted as the absolute risk difference between the highest and lowest educational levels.			
3. Estimates for the relative index of inequality, which was calculated using a log-binomial model, can be interpreted as the risk ratio between the highest and lowest educational levels.			

## Data Availability

All the data used in this study were obtained from the Ministries in Japan and can be provided if the Ministries approve.

## Supplementary Materials

The following supporting information can be downloaded at: https://www.mdpi.com/2227-9067/10/2/342/s1, Table S1: Results of slope index of inequality and relative index of inequality for preterm birth rate depending on parental educational levels when using an imputation method.
