# Correction: Okui, T. Analysis of an Association between Preterm Birth and Parental Educational Level in Japan Using National Data. *Children* 2023, *10*, 342

**DOI:** 10.3390/children10061034

**Published:** 2023-06-08

**Authors:** Tasuku Okui

**Affiliations:** Medical Information Center, Kyushu University Hospital, Fukuoka City 812-8582, Japan; okui.tasuku.509@m.kyushu-u.ac.jp; Tel.: +81-092-642-5881

## Error in Figure/Table

In the original publication [1], there was a mistake in Figure 1, Table 1, Table 2, Table 3 and Table S1 as published. In this study, one-to-one matching pairs between parents in birth data and men and women in the Census data from Japan were included in the study population via data linkage. Data linkage was conducted by writing programming codes using a statistical software. However, some of the many-to-one matching pairs were included in the study population because of programming errors by the author. Therefore, the author wishes to publish a result that corrects this error. The study population decreased from 782,536 to 777,086 after the correction, and the numeric values in the tables and figure need to be corrected accordingly. The corrected Figure 1, Table 1, Table 2, Table 3 and Table S1 appear below. The author states that the scientific conclusions are unaffected. This correction was approved by the Academic Editor. The original publication has also been updated.

## Text Correction

There was an error in the original publication [1]. The mistake is explained in the previous section. Corrections have been made to the Abstract, Materials and Methods, and Results. The original and corrected texts are provided below.
**Page****Original****Corrected**Page 1, Abstract, line 8782,536777,086Page 1, Abstract, line 95.09 and 5.205.07 and 5.21Page 2, Data Linkage, line 15782,536777,086Page 4, Results, lines 2–3311,050 in 2000 to 217,968 in 2020308,994 in 2000 to 216,637 in 2020Page 4, Results, line 115.09 and 5.205.07 and 5.21Page 4, Results, line 24−0.618−0.609Page 4, Results, line 280.8530.854

**Figure 1:**
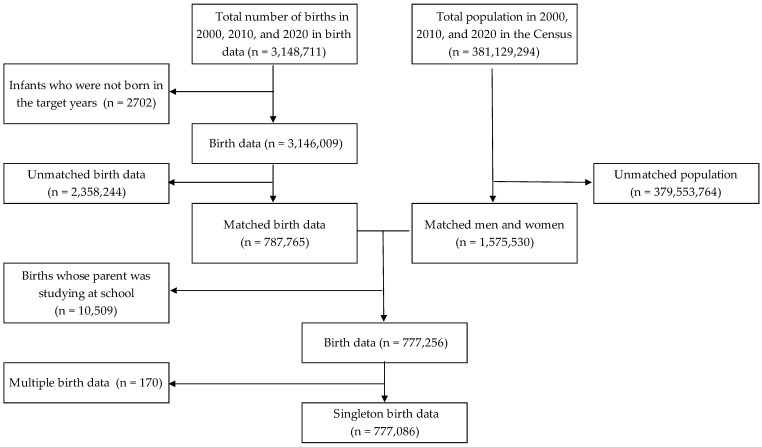
The flowchart of the data selection process.

**Table 1:** Number of births for each attribute by year.

	Year		
	2000	2010	2020
Total	308,994 (100.0)	251,455 (100.0)	216,637 (100.0)
Maternal age group			
19 years or less	9607 (3.1)	5076 (2.0)	2013 (0.9)
20–24 years	72,551 (23.5)	50,407 (20.0)	31,218 (14.4)
25–29 years	112,295 (36.3)	82,313 (32.7)	65,429 (30.2)
30–34 years	81,107 (26.2)	69,971 (27.8)	66,501 (30.7)
35–39 years	29,172 (9.4)	36,087 (14.4)	40,761 (18.8)
40 years or more	4262 (1.4)	7601 (3.0)	10,715 (4.9)
Gender			
Female	149,954 (48.5)	122,360 (48.7)	105,734 (48.8)
Male	159,040 (51.5)	129,095 (51.3)	110,903 (51.2)
Parity			
Primiparous	156,453 (50.6)	125,412 (49.9)	104,657 (48.3)
Multiparous	152,541 (49.4)	126,043 (50.1)	111,980 (51.7)
Household occupation			
Farmer	20,371 (6.6)	8193 (3.3)	4175 (1.9)
Self-employed	30,261 (9.8)	21,016 (8.4)	17,089 (7.9)
Full-time worker 1	116,984 (37.9)	96,872 (38.5)	75,969 (35.1)
Full-time worker 2	100,111 (32.4)	89,426 (35.6)	92,264 (42.6)
Other occupations	34,218 (11.1)	25,703 (10.2)	21,046 (9.7)
Unemployed	3624 (1.2)	3910 (1.6)	1721 (0.8)
Missing	3425 (1.1)	6335 (2.5)	4373 (2.0)
Paternal educational level			
Junior high school	36,536 (11.8)	21,616 (8.6)	13,555 (6.3)
High school	167,938 (54.3)	109,471 (43.5)	75,470 (34.8)
Technical school or junior college	34,399 (11.1)	34,600 (13.8)	27,607 (12.7)
University or graduate school	66,594 (21.6)	66,058 (26.3)	72,419 (33.4)
Missing	3527 (1.1)	19,710 (7.8)	27,586 (12.7)
Maternal educational level			
Junior high school	25,841 (8.4)	16,964 (6.7)	9896 (4.6)
High school	173,690 (56.2)	106,675 (42.4)	71,571 (33.0)
Technical school or junior college	83,233 (26.9)	72,275 (28.7)	54,595 (25.2)
University or graduate school	22,671 (7.3)	36,647 (14.6)	53,626 (24.8)
Missing	3559 (1.2)	18,894 (7.5)	26,949 (12.4)
Gestational age			
Term birth	294,936 (95.5)	239,867 (95.4)	206,784 (95.5)
Preterm birth	13,969 (4.5)	11,548 (4.6)	9821 (4.5)
Missing	89 (0.0)	40 (0.0)	32 (0.0)
Birthweight			
>= 2, 500 g	285,929 (92.5)	230,548 (91.7)	199,587 (92.1)
< 2500 g	23,042 (7.5)	20,876 (8.3)	17,023 (7.9)
Missing	23 (0.0)	31 (0.0)	27 (0.0)

**Table 2:** Preterm birth rate (%) by year and parental educational level.

	Year		
	2000	2010	2020
Total	13,597 (4.51)	10,246 (4.56)	8357 (4.52)
Paternal educational level			
Junior high school	1892 (5.27)	1045 (5.04)	686 (5.21)
High school	7446 (4.50)	4959 (4.68)	3366 (4.57)
Technical school or junior college	1439 (4.24)	1456 (4.33)	1187 (4.39)
University or graduate school	2820 (4.28)	2786 (4.32)	3118 (4.39)
Maternal educational level			
Junior high school	1397 (5.52)	854 (5.28)	488 (5.07)
High school	7834 (4.58)	4845 (4.72)	3248 (4.70)
Technical school or junior college	3438 (4.18)	3055 (4.35)	2388 (4.45)
University or graduate school	928 (4.13)	1492 (4.16)	2233 (4.24)

**Table 3:** Results of the slope index of inequality and relative index of inequality for the preterm birth rate depending on parental educational level.

	2000	2010	2020
	Estimates (95%CI)	Estimates (95%CI)	Estimates (95%CI)
Slope index of inequality			
Paternal educational level	−0.609 (−0.924, −0.293)	−0.620 (−0.976, −0.264)	−0.489 (−0.876, −0.103)
Maternal educational level	−1.024 (−1.344, −0.705)	−1.061 (−1.422, −0.700)	−0.967 (−1.353, −0.580)
Relative index of inequality			
Paternal educational level	0.854 (0.795, 0.918)	0.867 (0.800, 0.939)	0.886 (0.812, 0.967)
Maternal educational level	0.779 (0.723, 0.838)	0.773 (0.713, 0.839)	0.784 (0.719, 0.856)
CI, confidence intervals			
1. Gender, parity, household occupation, and maternal age group were adjusted in the analysis.			
2. Estimates for the slope index of inequality, which was calculated using a binomial model with an identity link function, can be interpreted as the absolute risk difference between the highest and lowest educational levels.			
3. Estimates for the relative index of inequality, which was calculated using a log-binomial model, can be interpreted as the risk ratio between the highest and lowest educational levels.

**Table S1:** Results of the slope index of inequality and relative index of inequality for the preterm birth rate depending on parental educational level using an imputation method.

	2000	2010	2020
	Estimates (95%CI)	Estimates (95%CI)	Estimates (95%CI)
Slope index of inequality			
Paternal educational level	−0.602 (−0.913, −0.290)	−0.542 (−0.879, −0.206)	−0.496 (−0.851, −0.141)
Maternal educational level	−0.975 (−1.291, −0.660)	−0.986 (−1.329, −0.644)	−0.734 (−1.092, −0.377)
Relative index of inequality			
Paternal educational level	0.855 (0.796, 0.918)	0.882 (0.818, 0.950)	0.885 (0.817, 0.959)
Maternal educational level	0.788 (0.733, 0.847)	0.789 (0.731, 0.852)	0.832 (0.767, 0.901)
CI, confidence intervals			
1. Gender, parity, household occupation, and maternal age group were adjusted in the analysis.			
2. Estimates for the slope index of inequality, which was calculated using a binomial model with an identity link function, can be interpreted as the absolute risk difference between the highest and lowest educational levels.			
3. Estimates for the relative index of inequality, which was calculated using a log-binomial model, can be interpreted as the risk ratio between the highest and lowest educational levels.			
