# Supplementary material for: Analysis of an Association between Preterm Birth and Parental Educational Level in Japan Using National Data
Source: Children (Basel). 2023 Feb 9;10(2):342. doi: 10.3390/children10020342 (PMC9954840; doi:10.3390/children10020342)
Supplement: Supplementary file 1 [file children-10-00342-s001.zip › children-2158177 just supplementary correction.pdf]

Table S1. Results of slope index of inequality and relative index of inequality for preterm birth rate depending on parental educational levels when using an imputation method.

	2000	2010	2020
	**Estimates (95%CI)**	**Estimates (95%CI)**	**Estimates (95%CI)**
Slope index of inequality			
Paternal educational level	−0.602 (−0.913, −0.290)	−0.542 (−0.879, −0.206)	−0.496 (−0.851, −0.141)
Maternal educational level	−0.975 (−1.291, −0.660)	−0.986 (−1.329, −0.644)	−0.734 (−1.092, −0.377)
Relative index of inequality			
Paternal educational level	0.855 (0.796, 0.918)	0.882 (0.818, 0.950)	0.885 (0.817, 0.959)
Maternal educational level	0.788 (0.733, 0.847)	0.789 (0.731, 0.852)	0.832 (0.767, 0.901)

CI, confidence intervals

1. Gender, parity, household occupation, and maternal age group were adjusted in the analysis.
2. Estimates for slope index of inequality, which was calculated by binomial model with identity link, can be interpreted as absolute risk difference between the highest and the lowest educational levels.
3. Estimates for relative index of inequality, which was calculated by log-binomial model, can be interpreted as risk ratio between the highest and the lowest educational levels.
